# Assessing the Intention to Use Sports Bracelets Among Chinese University Students: An Extension of Technology Acceptance Model With Sports Motivation

**DOI:** 10.3389/fpsyg.2022.846594

**Published:** 2022-03-23

**Authors:** Yi Wang, Xiaotian Zhang, Li Wang

**Affiliations:** ^1^Faculty of Education, University of Macau, Taipa, Macao SAR, China; ^2^School of Psychology, Beijing Sport University, Beijing, China

**Keywords:** technology acceptance model, intention to use technology, sports bracelet, sports motivation, university students

## Abstract

The use of mobile technology, such as sports bracelets, is gaining popularity as it modifies the ways and processes of learning and teaching in college physical education (PE). However, little empirical evidence can be seen in literature to demonstrate crucial factors that influence university students’ acceptance of sports bracelets. Guided by the technology acceptance model (TAM), this study hence aimed at explaining university students’ intention to use sports bracelets. In total, 445 university students in China responded to a 19-item survey package. Results indicated that users’ attitudes toward technology and perceived usefulness were significant predictors of intention to use sports bracelets. Meanwhile, users’ intrinsic sports motivation significantly promoted users’ positive attitudes toward this technology and was significantly influenced by perceived ease of use and perceived usefulness. Overall, our findings highlighted the importance of stimulating young adults’ intrinsic sports motivation that facilitates their intention to use sports bracelets such that to develop a healthy lifestyle that benefits their physical health.

## Introduction

Mobile technology has been permeating most aspects of university students’ lives over the past decade. Alongside the integration of mobile technology with educational purposes into higher education, advances in such technology, and the availability of associated cyber sources avail the opportunities for ubiquitous and flexible learning to users (Pimmer et al., 2016). Empirical evidence signaled that notwithstanding the direct impact of mobile technology on effective learning ways and processes, university students’ intention to use certain mobile technological products can be influenced by technology infrastructure (Kim et al., 2017), users’ satisfaction (Du, 2015), instructional support for mobile learning (Safford and Stinton, 2016), and users’ technology literacy (George and DeCristofaro, 2016). Insofar as the reliance on mobile technology grows for preparing students for the twenty-first century workplace, identifying motivators and hinders related to learners’ acceptance of mobile technology have been at the foci of vibrant and proliferating streams of research across cultures and disciplines (e.g., Chang et al., 2017; Hanafi et al., 2018; Saroia and Gao, 2019; Hoi, 2020). As Teo and Zhou (2014) pointed out, it remains an important issue to understand how learners accept and use a technological product.

Alongside the globally recognized importance to promote the health of young adults who have been spending a considerable amount of time in sedentary behavior (Peterson et al., 2018), mobile technology improves physical health-related quality of life mushrooms in the marketplace. Sports bracelet is one of such products known for their wearability along with powerful data collection and analysis capabilities (Khare, 2017). Huang and Wang (2018) have given accolades to sports bracelet for its feasibility to be applied in college physical education (PE) with its five key functions: function of fitness, the function of activity record, function of social networking analysis, function of exercise promotion, and function of 24-h and 7-day monitoring. Given its personalized nature stressing individual characteristics, such as body mass index, sports habits, and preferences, they further observed the increasing popularity yet still peripheral use of sports bracelets among university students in physical activities inside and outside PE classes. Researchers have endeavored to investigate the effectiveness of sports bracelets in monitoring different types of physical activity and denoted their power in stimulating interests in sports, strengthening subjective consciousness, and promoting sports abilities (e.g., Weghorn, 2016; Hao et al., 2019). Limited empirical evidence, however, can be found in literature deciphering individuals’ perceptions of and intention to use this technology for fitness improvement. This warranted the investigation of university students’ acceptance of sports bracelets, which has great potential to make their learning, sports, rest, and diet to achieve a more scientific balance. The present study, therefore, made an early attempt to identify essential factors that affected university students’ intention to use sports bracelets.

### Literature Review

#### Technology Acceptance Model

User acceptance is a crucial factor for successful technology implementation. Originated from the theory of reasoned action (TRA) and the theory of planned behavior (TPB), the technology acceptance model (TAM) has been widely acknowledged and used to understand technology users’ potential acceptance or rejection of a technology (Davis, 1989; Marangunić and Granić, 2015). Two determinants are identified in predicting users’ technology acceptance: perceived ease of use and perceived usefulness. Perceived ease of use refers to “the degree to which a person believes that using a particular system would be free of effort” and perceived usefulness is defined as “the degree to which a person believes that using a particular system would enhance his or her job performance” (Davis, 1989, p. 428). The two factors together with attitudes, an individual’s positive or negative feelings about performing the action, can impact individuals’ intention to use technology (Davis, 1989; Marangunić and Granić, 2015).

Although it has been widely accepted and used, TAM still has some limitations, among which lacking of motivational constructs is a major concern (e.g., Davis et al., 1992; Venkatesh et al., 2003; Pedrotti and Nistor, 2016). In view of this, several researchers have attempted to enrich TAM by emphasizing and adding motivational factors across disciplines (Fagan et al., 2008; Sánchez and Hueros, 2010; Zhou, 2016; Huang, 2017; Nikou and Economides, 2017). However, the main concepts and constructs in motivational models usually overlapped with those in TAM by regarding perceived enjoyment as intrinsic motivation and taking perceived usefulness as extrinsic motivation (EM). To this end, an expansion of incorporating motivational factors in TAM was advocated for better interpretation of how other factors can be added to the core TAM variables “to achieve greater explanatory powers and validity” (Teo and Zhou, 2014, p. 127).

#### Intrinsic and Extrinsic Sports Motivation

Motivation is a hot topic in the PE or sports domain, which elicited researchers’ efforts to investigate its functions and effects on individuals’ behaviors, persistence, learning, and performance (e.g., Pelletier et al., 1995, 2013; Lee et al., 2017). To extend the measurement of sports motivation into a broader context, Pelletier et al. (2013) validated the Sport Motivation Scale (SMS-II) that presented a tripartite intrinsic-extrinsic motivation taxonomy based on the self-determination theory (Ryan and Deci, 2000). Intrinsic sports motivation refers to individuals’ engagement in a physical activity purely for the pleasure and satisfaction derived from doing the activity *per se*, which consists of three subcategories: intrinsic motivation to know, intrinsic motivation to accomplish things, and intrinsic motivation to experience stimulation. Extrinsic sports motivation pertains to multiple engaging physical actions and behaviors as means to goal achievement (Deci, 1975; Pelletier et al., 2013). It has been classified into three categories: external regulation, introjection, and identification. They lie along the self-determination continuum from lower to higher levels (Pelletier et al., 2013). In the sports domain, the more self-determined types of motivation contributed more to sports participation intensity, sports persistence, affective feelings of sports, interests in sports, and satisfaction toward sports, or sports dropout possibility (e.g., Martens and Webber, 2002; Standage et al., 2003; Lee et al., 2017). Sports motivations were also significant predictors of activity intentions. Standage et al. (2003) explored secondary school students’ intention to partake in leisure-time physical activities, results revealed a positive relationship between self-determined motivation and activity intention.

Sports bracelet is an emerging activity-tracking product that is able to initiate necessary inner drives for physical activities (Mercola, 2016; Donnachie et al., 2017). As a technology with the potential to stimulate users’ different types of motivations for physical actions, however, it has received limited attention on its associations with sports motivations. To this end, we drew on the TAM and the intrinsic-extrinsic-tripartite sports motivational frameworks to investigate essential factors that influence students’ intention to use sports bracelets in their physical activities.

The purpose of this study was to establish an extended TAM model and evaluate its exploratory potential among a group of Chinese students in tertiary institutions. We extended TAM with both intrinsic and extrinsic sports motivation to investigate influential factors for students’ intention to use sports bracelets in physical activities. We selected two sub-constructs of sports motivation: intrinsic motivation to know and identification of extrinsic motivation based on the following considerations that given the benefits of sports bracelets in promoting physical activities, the two constructs are at a higher self-determined level, which could enhance psychological functioning and elicit adaptive motivational responses and in turn stimulate more actions (Standage et al., 2003). Taken together, we developed five hypotheses to uncover the relations between students’ sports motivations and their intention to adopt sports bracelets.

### Hypothesis Development

#### Attitudes

Studies have strengthened the vital role of attitudes in predicting learner’s intention to use technology (Davis, 1989; Hoi, 2020). Previous studies have repeatedly shown that there was a significant relationship between attitudes and intention to use wearable devices, such as smartwatches (Choi and Kim, 2016; Lunney et al., 2016). Accordingly, in the current study, we also assumed that positive attitudes can drive individuals to use sports bracelets and vice versa. Thus, the first hypothesis was proposed:

**H1** Students’ attitudes toward sports bracelets will have a significant influence on their intention to use the device.

#### Perceived Usefulness

Perceived usefulness was found to have a direct influence on the attitudes toward technology and had a positive impact on behavioral intention to use technology by the mediation effect of attitudes toward technology (Davis et al., 1992; Venkatesh et al., 2003; Choi and Kim, 2016; Lunney et al., 2016). In a TAM study, which explored taekwondo competitors’ acceptance of electronic body protectors and scoring systems, perceived usefulness was significantly related to attitudes and further influenced their purchasing intention (Ko et al., 2011). Perceived usefulness, as a key driver concerning utility values for technology usage, was also found to significantly correlate with intrinsic motivations to use technology (Lee et al., 2005; Yoo et al., 2012). Theoretically, perceived usefulness relates to the attitudes and intention to use technology (i.e., sports bracelets). The usage of sports bracelets, in turn, had the potential to facilitate students’ autonomy, competence, and relatedness to be intrinsically motivated to adopt sports bracelets (Murcia et al., 2009). Such inclination for technology adoption can deepen students understanding of sports bracelets and facilitate their ultimate goals in using sports bracelets for physical development. Therefore, the increase of autonomy, competence, and relatedness of sports bracelets may also be able to cultivate students’ intrinsic motivation to sports bracelets and their intrinsic sports motivation, which can further influence their attitudes toward sports bracelets and their intentions to use them (Ntoumanis, 2005; Murcia et al., 2009). To wit, if users perceived that sports bracelet is useful and beneficial, they would not only be more intrinsically motivated to learn, accept, and utilize it, but also more likely to hold positive attitudes toward sports. To this end, we hypothesized that:

**H2** Students’ perceived usefulness of sports bracelets will have a positive significant influence on their (a) intrinsic sports motivation, (b) attitudes toward sports bracelets, and (c) intention to use sports bracelets.

#### Perceived Ease of Use

According to TAM, perceived ease of use has a significant influence on perceived usefulness (Davis et al., 1992; Venkatesh et al., 2003; Choi and Kim, 2016). If users perceive that using the technology is free of effort, they tend to recognize the usefulness of the technology. Further, informed by the positive association between perceived ease of use and attitudes toward the intention to use devices or systems (e.g., Wu et al., 2016; Nikou and Economides, 2017), perceived ease of use can also influence students’ intention to use devices as mediated by their attitudes. The impact of perceived ease of use on acceptance of technology, theoretically underpinned by TAM and self-determination theory can also be extended to sports motivation in PE (Davis et al., 1992; Ntoumanis, 2005; Murcia et al., 2009). According to Ryan and Deci (2000), perceived ease of use can be associated with intrinsic sports motivation. If students perceive that using sports bracelets is free of effort, they will have a tendency to accept and use sports bracelets in their sports practices, through which they can gain accumulated experience and competence of it, understand the benefits of sports bracelets in sports practices, and be more motivated to participate in the sports practices on their own for the sake of personal development. Taken together, we proposed the third hypothesis:

**H3** Students’ perceived ease of use will have a positive significant influence on their (a) perceived usefulness, (b) attitudes toward technology, (c) intrinsic sports motivation, (d) extrinsic sports motivation, and (e) intention to use sports bracelets.

#### Intrinsic and Extrinsic Sports Motivation

Intrinsic sports motivation refers to students’ practicing sports for the pleasure and the satisfaction that they experience while learning, exploring, or trying to understand it (Pelletier et al., 2013). Intrinsic motivation in sports domain is crucial for individuals’ persistence, positive emotions, greater interest, and sports satisfaction (Pelletier et al., 1995, 2013; Standage et al., 2003). In other words, such motivations are able to induce more positive consequences and lead to more enjoyable feelings (Murcia et al., 2009). Given this relationship, we assumed that students who are more intrinsically motivated to wear sports bracelets in their sports practices for sheer pleasure will have more positive attitudes toward sports bracelets. We hence proposed the fourth hypothesis:

**H4** Students’ intrinsic sports motivation will have a significant influence on their attitudes toward sports bracelets.

As a contributing factor to behavioral persistence, extrinsic sports motivation, identified with a higher autonomous level on the controlled-to-autonomous motivation continuum, has the potential to predict intention to use technology (e.g., Ryan and Deci, 2000). That is, students who are more extrinsically motivated to wear sports bracelets in sports practices due to their improved knowledge and recognition of the technology will better appreciate the benefits of this technology, and will be more internally self-regulated and self-determined to use this technology in their practices (Pelletier et al., 2013). Meanwhile, as indicated by self-determination theory (Ryan and Deci, 2000), extrinsic sports motivation can also be influenced by perceived ease of use. If students perceive it is difficult to accept or utilize sports bracelets in their sports practices, they may weaken the importance of the technology and its usefulness in attaining their personal goals. Consequently, they can be less extrinsically motivated to accept sports bracelets in sports practices. Accordingly, the fifth hypothesis was developed as follows:

**H5** Students’ extrinsic sports motivation will be significantly influenced by perceived ease of use, and students’ extrinsic sports motivation will significantly influence intention to use technology.

**FIGURE 1:**
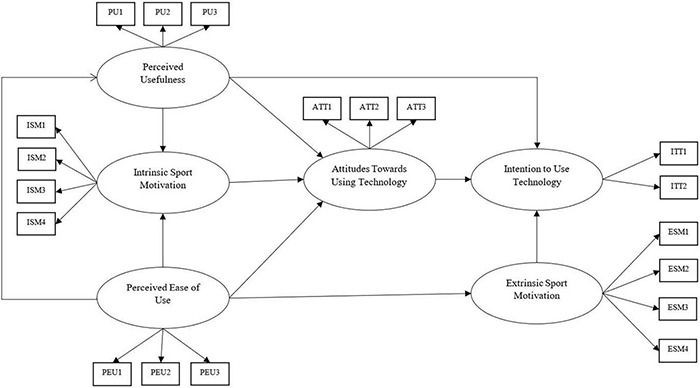
Hypothesized model (permission for using TAM in the current research has been obtained from the authors).

The hypothesized model is presented in Figure 1.

## Method

### Participants

In total, 445 participants were invited to complete the online survey on a voluntary basis (50.6% were women). The average age was 19.05 [standard deviation (SD) = 1.59]. Of the 445 students, 84.5% majored in Social Science and Humanities and 15.5% majored in Science and Technology. Invitations to participate in this study with an online survey link or quick response (QR) code were sent out to 503 WeChat (a popular online social networking platform in China) users who were currently college students. In total, 58 cases that reported they have never used any type of sports bracelets were removed, leading to a final sample of 445.

### Measures

The survey items were adapted from previous studies that were found to be statistically reliable (Davis, 1989; Pelletier et al., 2013). The six constructs presented in the research model were measured by 19 items: intrinsic sports motivation (4 items); extrinsic sports motivation (4 items); perceived usefulness (3 items); perceived ease of use (3 items); attitudes toward using technology (3 items); and intention to use technology (2 items). Each item was rated on an 11-point Likert scale, ranging from 0 (strongly disagree) to 10 (strongly agree). Participants’ demographic information (e.g., gender, age, and majors) was also collected. A full list of survey items was presented in the “Appendix A.”

### Data Analysis

As reminded by Anderson and Gerbing (1988), when assessing model fit, comparison between the target model and other models should be ensured. Therefore, a standard two-step approach to structural equation modeling (SEM) was used in the present study. The first step was to conduct a confirmatory factor analysis (CFA) to examine the validity of constructs within the measurement model. The measurement model provides a baseline comparison for the structural model and an upper limit on the fit of the saturated variable model (Baumgartner and Weijters, 2010). In the second step, SEM was performed to test the proposed model using the maximum likelihood estimation (MLE) method in Amos 23.0.

## Results

### Evaluation of the Measurement Model

The mean values of all 19 items were above the mid-point of 5.0, ranging from 5.98 to 7.18. The SDs ranged from 1.49 to 1.86, revealing an overall positive response to all items that were used to measure the constructs within the model, and a fairly narrow spread of scores around the mean (see Table 1). Positive correlations among the six constructs were identified. The values of skewness (ranged from − 0.02 to 0.08) and kurtosis (ranged from −0.90 to −0.74) were between the recommended cutoffs from the six constructs were identified. univariate normality of data (Kline, 2010). Hoelter’s (1983) critical N was valued by researchers to ensure reliable results in SEM (Callum et al., 2014; Hoque and Sorwar, 2017). The sample size of this study is 445, and Hoelter’s (1983) critical N for the model is 264, indicating the hypothesis that the proposed model is correct would be accepted at a 0.01 level of significance. Therefore, SEM was considered as an appropriate technique for data analysis.

**TABLE 1:** Means, standard deviations, and correlations of the constructs.

	Constructs	1	2	3	4	5	6
1	Perceived usefulness (PU)	1					
2	Perceived ease of use (PEU)	0.49**	1				
3	Attitudes toward using technology (ATT)	0.63**	0.64**	1			
4	Intention to use technology (ITT)	0.44**	0.44**	0.53**	1		
5	Intrinsic sport motivation (ISM)	0.34**	0.32**	0.38**	0.25**	1	
6	Extrinsic sport motivation (ESM)	0.31**	0.29**	0.30**	0.23**	0.70**	1
	Mean	6.98	6.79	6.91	6.48	6.87	6.31
	SD	1.6	1.49	1.39	1.53	1.39	1.34

**p < 0.01.

The MLE procedure that assumes multivariate normality of the observed variables was employed to assess the measurement model of the present study. According to Mardia’s (1970) and Raykov and Marcoulides (2008) normalized multivariate kurtosis value should be lower than *p* (*p*+2), where *p* means the number of observed variables in the model. The Mardia’s coefficient in this study was 194.22, lower than 399 [19 × (19 + 2)]. Therefore, multivariate normality of the data was assumed.

Average variances extracted (AVE) and composite reliability (CR) were employed to evaluate the validity and reliability of internal constructs. Compared to Cronbach’s α, AVE and CR are considered to better comply with the key assumptions in the multidimensional scale (Teo and Fan, 2013). Factor loadings of all the items in the measuring range from 0.65 to 0.98 meeting the threshold suggested by Hair et al. (2010) that an item is significant if its factor loading is above 0.50. The AVE, a more conservative indicator of validity, was above 0.4. The CR was above 0.6. Both values of AVE and CR were hence considered to be statistically acceptable for being above 0.4 (Fornell and Larcker, 1981; Hair et al., 2010). As shown in Table 2, the standardized factor loading, AVE, and CR of all constructs met the aforementioned guidelines, which jointly indicated that the measures were reliable.

**TABLE 2:** Results of the measurement model, composite reliability (CR), and average variance extracted (AVE).

Construct	Item	Standardized factor loading	*t*-value	CR^a^	AVE^b^
Intention to use (ITT)	ITT1	0.699	–	0.836	0.724
	ITT2	0.979	12.361***		
Attitudes toward using technology (ATT)	ATT1	0.884	–	0.907	0.765
	ATT2	0.893	25.998***		
	ATT3	0.847	23.692***		
Perceived usefulness (PU)	PU1	0.909	–	0.929	0.813
	PU2	0.898	28.720***		
	PU3	0.898	28.717***		
Perceived ease of use (PEU)	PEU1	0.796	–	0.907	0.766
	PEU2	0.915	22.157***		
	PEU3	0.910	22.061***		
Intrinsic sport motivation (ISM)	ISM1	0.740	16.803***	0.863	0.612
	ISM2	0.810	18.822***		
	ISM3	0.755	17.221***		
	ISM4	0.821	–		
Extrinsic sport motivation (ESM)	ESM1	0.697	–	0.783	0.491
	ESM2	0.686	12.627***		
	ESM3	0.719	13.148***		
	ESM4	0.650	12.043***		

***p < 0.001. $a\frac{{\left(\sum \lambda \right)}^{2}}{{\left(\sum \lambda \right)}^{2}+\left(\sum 1-\lambda^{2}\right)}.$$b\frac{\sum \lambda^{2}}{\sum \lambda^{2}+\left(\sum 1-\lambda^{2}\right)}.$

The following indices were adopted to test the model fit within SEM: goodness-of-fit index (GFI), comparative fit index (CFI), and Tucker-Lewis index (TLI). As recommended by researchers (Hair et al., 2010), a value of 0.9 and higher of these indices can be considered adequate. In addition to the root-mean-square error of approximation (RMSEA), the standardized root mean square residual (SRMR), with a value of <0.08, indicate that an acceptable fit was used (Hu and Bentler, 1999). Results of SEM analysis suggested a good fit between the measurement model and the whole dataset: *χ^2^* = 286.61, *χ^2^*/df = 2.09, GFI = 0.94, CFI = 0.97, TLI = 0.97, RMSEA = 0.050, and SRMR = 0.036.

### Evaluation of the Structural Model and Hypothesis Testing

**FIGURE 2:**
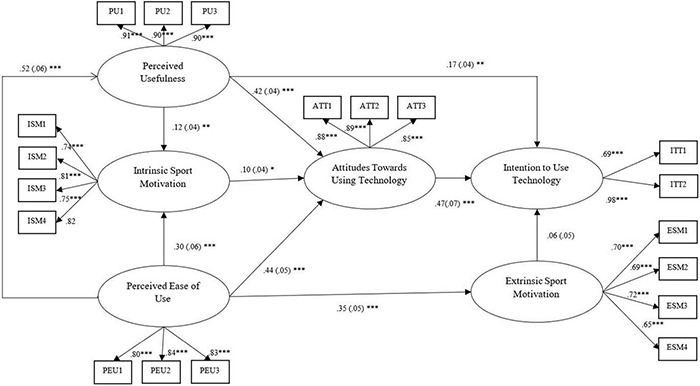
The path coefficients. *p < 0.05; **p < 0.01; ***p < 0.001.

Results of the hypothesis testing, structural model, and path coefficients are summarized in Table 3 and Figure 2. The results reflected the relationship among the constructs regarding their magnitudes and significance, from which, each of the hypotheses can be decided to be either supported or rejected. The structural model had a good fit: *χ^2^* = 308.270, *χ^2^*/df = 2.19, GFI = 0.93, CFI = 0.97, TLI = 0.96, RMSEA = 0.052, and SRMR = 0.052. All but one hypothesis (H5) was supported by the results. Hypotheses 1–3 were significant, supporting the TAM as a valid framework in explaining students’ intention to use sports bracelets. Of the external constructs, intrinsic sports motivation was significantly affected by perceived usefulness and perceived ease of use. Intrinsic sports motivation, on the other hand, exerted significant influence only on the attitudes toward technology, by which H4 was supported. In addition, the extrinsic sports motivation was significantly influenced by perceived ease of use but could not predict the intention to use sports bracelets, partially supporting H5.

**TABLE 3:** Hypothesis testing.

Hypothesis	Path	Path coefficient	*t*-value	Results
H1	Attitudes toward technology → Intention to use technology	0.471	6.428***	Support
H2	Perceived usefulness → Intrinsic sport motivation	0.118	2.637**	Support
	Perceived usefulness → Attitudes toward technology	0.331	9.337***	Support
	Perceived usefulness → Intention to use technology	0.121	2.860**	Support
H3	Perceived ease of use → Intrinsic sport motivation	0.308	5.228***	Support
	Perceived ease of use → Attitudes toward technology	0.449	9.051***	Support
	Perceived ease of use → Perceived usefulness	0.522	10.538***	Support
H4	Intrinsic sport motivation → Attitudes toward technology	0.104	2.582*	Support
H5	Extrinsic sport motivation → Intention to use technology	0.054	1.213	Not support
	Perceived ease of use → Extrinsic sport motivation	0.334	6.190***	Support

***p < 0.001, **p < 0.01, *p < 0.05.

From the final model (see Figure 2), five endogenous constructs were tested. Of the variance in intention to use technology, 30.1% was explained by attitudes toward technology, perceived usefulness, and extrinsic sports motivation. The attitude toward using technology was significantly predicted by perceived usefulness, perceived ease of use, and intrinsic sports motivation with an *R*^2^ of 0.544, demonstrating the three constructs could explain 54.4% of the variance in attitudes toward technology. Of the variance in intrinsic sports motivation, 14.7% was significantly explained by perceived ease of use and perceived usefulness. The variances in the other two endogenous constructs, perceived usefulness and extrinsic sports motivation, were explained by the determinant constructs in amounts of 24.2 and 8.6%, respectively.

## Discussion

The aim of this study was to enrich the TAM by introducing refined motivational factors (i.e., intrinsic motivation to know and identification in extrinsic motivation) into technology acceptance and assess the predictors of college students’ intention to use sports bracelets. Results indicated that perceived usefulness, perceived ease of use, and sports motivation significantly predicted students’ attitudes toward sports bracelet use, wherein 61.9% of the variance was explained. Students’ intention to use sports bracelets was influenced by their perceived usefulness and attitudes toward sports bracelet use, supporting H1–H3. The findings echoed previous TAM research, which suggested that perceived usefulness and attitudes were important factors that influenced students’ intention to use a technology (Schepers and Wetzels, 2007; Zhang et al., 2012; Teo and Zhou, 2014).

In this study, intrinsic sports motivation and extrinsic sports motivation, as the external constructs of the TAM, were additionally examined. Rooted in self-determination theory, this study identified predictive factors of individuals’ behavioral intention to use sports bracelets. The hypotheses (H4) related to sports motivation were supported, but the hypothesis (H5) involving extrinsic sports motivation was not supported as the path from extrinsic sports motivation to intention to use the technology was not significant. The results were out of line with Fagan et al.’s (2008) study in which EM was found to positively predict first-line managers’ intention to use computers. In the setting of higher education, EM was also reported to significantly predict students’ intention to use the Internet-based learning medium (Lee et al., 2005). However, our results were consistent with Yoo et al.’s (2012) findings, which denoted that the extrinsic motivators did not directly promote e-learning at the workplace in South Korea.

Several plausible explanations need to be noted for the inconformity between the findings. First, although scholars have widely recognized the important role of motivation in TAM (Atkinson, 1997; Venkatesh and Davis, 2000), it is possible that the inconsistency between findings of this study and others was caused by the differences in motivational constructs and sports technologies of investigation (Pedrotti and Nistor, 2016; Zhou, 2016; Nikou and Economides, 2017). Second, the effect size of intrinsic and extrinsic sports motivation might be different in terms of individuals’ behavioral intention to use technology. As Huang (2017) has suggested that the influence of intrinsic motivation on intention to use sports bracelets was larger than that was exerted by extrinsic motivation. Compared with extrinsic sports motivation, intrinsic sports motivation was proved to more closely relate to individuals’ perceptions of competence, autonomy, and levels of self-determination (Ryan and Deci, 2000; Murcia et al., 2009). Third, extrinsic sports motivation can be determined by external sources that impact an individual’s behavior involved in sports, such as motivational climate (Standage et al., 2003), supports from friends, parents, and material rewards (Gordon et al., 1995). Given that extrinsic sports motivation can be influenced by other contextual factors (e.g., external rewards), Ruskin et al. (2007) found that individuals who performed sports to improve themselves were more likely to remain motivated than those who practiced merely for gaining rewards. In other words, students who perform sports for external rewards may have a low level of desire for adopting sports equipment, such as wearing bracelets, in a bid to improve their fitness. In the current study, sports performed by the students were not associated with any rewards, the extrinsic sports motivation can therefore be reduced in non-voluntary environments (Wu and Lederer, 2009), that is, the power of extrinsic sports motivation in predicting students’ intention to use bracelet could have been reduced. Fourth, the missing relationship between extrinsic sports motivation and intention to use sports bracelets may imply that individuals who were inclined to use this technology were driven by their beliefs, attitudes, and intrinsic motivation, but barely directly guided by extrinsic sports motivation. The influence of extrinsic motivation on intention to use technology, as noted by Yoo et al. (2012), can be mediated by intrinsic motivation.

Despite the variety of research findings, the results of this study have a unique contribution to explaining students’ intention to use wearable technology from the perspective of sports motivation in higher education. TAM has been widely opined as a solid base theory for examining users’ intention to use wearable devices. Nonetheless, some researchers critiqued TAM for insufficiently explaining users’ technology adoption behaviors (Lin et al., 2007). Our study hereby responded to the call made by scholars (Conner and Armitage, 1998; Perugini and Bagozzi, 2001) and extended TAM by supplementing additional constructs in the context of PE. This is believed to proffer empirical evidence in promoting the probing of psychological processes involved in individuals’ perceptions of the value of a technology.

Following the significant influence that perceived usefulness has on intrinsic sports motivation, attitudes toward technology, and intention to use technology, educators would be suggested to focus on aspects of their course design in ways that promote utilitarian activities by highlighting the effective features and powerful capabilities of sports bracelet. From the results, attitudes toward technology and perceived usefulness significantly and directly influenced students’ intention to use sports bracelets. This requires a coherent strategy to stress the role of mobile technology in different procedures of teaching and learning during PE classes (i.e., lecture giving, interactions, administration, assessment, and feedback), as well as off-campus physical activities wherein students can personally experience effective learning process, and witness productive outcomes after harnessing the power of this mobile technology. Specific examples include efficiency in completing learning tasks, quick and convenient access to information for assessing physical ability and quality, and timely and personalized plans for improvement based on monitoring data. Meanwhile, to cultivate learners’ positive attitudes toward using sports bracelets, it is crucial to proffer them with adequate support and instruction for proceeding with mobile learning with this technology. Moreover, the use of sports bracelets could be a possible solution to a sedentary lifestyle among young adults. The findings of this study suggest that designers of sports bracelets can provide users with some practical functions that may strengthen their perceived usefulness and positive attitudes toward the technology, as it would in turn increase their intention to use sports bracelets. For example, the combination of heart rate monitoring and positioning can be used to detect whether the user is in a sedentary state. If so, reminding the user to stand up and stretch the muscles after sitting for a while and presenting example body movements on the app interface for the users to follow are believed as helpful to promote individuals’ healthy lifestyle. It is also a possible direction for future studies to explore whether the usage of sports bracelets directly develops individuals’ physical condition and active lifestyle.

Some limitations should be noted. First, this study has concentrated on collecting data only from university students that are familiar with the use of mobile technology and innovations in technology. Interpretation of the results hence needs to be careful in wider user market who are potential sports bracelet users. As compared with the younger generation, older adults often have weaker intentions to adopt new technology (Czaja et al., 2006; Wu et al., 2015). Future studies hence are encouraged to involve participants from more various age groups to further investigate the role that sports motivation plays in the acceptance and use of wearable devices. Second, the participants of this study share the same cultural background. To further validate the findings, students with different cultural backgrounds may be included in the sample. Third, the results of this study are based on the analysis of self-reported data. Future studies may include observational data to triangulate the findings and overcome the shortcomings of self-reported data.

## Conclusion

To conclude, the findings enriched the existing literature on critical factors that influence mobile technology use in PE by confirming with a Chinese sample that the intention to use sports bracelets can be determined particularly by users’ attitudes toward technology and perceived usefulness. Additionally, users’ intrinsic sports motivation plays a facilitating role in developing users’ positive attitudes toward sports bracelets, which in turn, can be significantly affected by perceived ease of use and perceived usefulness.

The current study made an early attempt and contributed empirical evidence to explain university students’ intention to use sports bracelets in college PE. The finite operationalization of perceived usefulness invites future studies to focus not only on primary fitness-related functions (e.g., pedometer function combined with body mass index through analysis of walking step spacing and energy consumption) but also on other relatively secondary functions, such as entertainment and social networking. As it has been repeatedly reported by previous studies (e.g., Venkatesh, 2014; Salimon et al., 2017) that users’ hedonic motivation could significantly determine the continued use of technology. Methodologically, future research into sports bracelet intention is suggested to include qualitative techniques such that outstanding features, desired, and undesired features perceived by users can be identified to enlighten technology developers who aim at tailoring products appealing to different potential users. Intervention studies that examine the effectiveness of sports bracelets in improving users’ physical quality will also be valuable. With such evidence that underscores the most effective functions of sports bracelets in fitness enhancement, educators will be able to make the best use of such features in their pedagogical practices to optimize the use of this technology in PE.

## Data Availability Statement

The original contributions presented in the study are included in the article/supplementary material, further inquiries can be directed to the corresponding author.

## Ethics Statement

The studies involving human participants were reviewed and approved by the Ethics Board of the School of Psychology, Beijing Sport University. The patients/participants provided their written informed consent to participate in this study.

## Author Contributions

YW, XZ, and LW contributed to the conception and structure of the manuscript and wrote the manuscript. XZ carried out the data collection. YW conducted the data analysis. All authors contributed to the article and approved the submitted version.

## Conflict of Interest

The authors declare that the research was conducted in the absence of any commercial or financial relationships that could be construed as a potential conflict of interest.

## Publisher’s Note

All claims expressed in this article are solely those of the authors and do not necessarily represent those of their affiliated organizations, or those of the publisher, the editors and the reviewers. Any product that may be evaluated in this article, or claim that may be made by its manufacturer, is not guaranteed or endorsed by the publisher.

## Figures and Tables

**TABLE 1:** Survey items.

Construct	Item	
Intention to use (ITT)^a^	ITT1	I intend to use bracelet for activities.
	ITT2	I will reuse bracelet for relevant activities.
Attitudes toward using technology (ATT)^a^	ATT1	Using bracelet is a good idea.
	ATT2	Using bracelet is a wise idea.
	ATT3	I like the idea of using bracelet.
Perceived usefulness (PU)^a^	PU1	Using bracelet in my sport engagement would enable me to accomplish tasks more quickly.
	PU2	Using bracelet would improve my sport performance.
	PU3	Using bracelet sport would increase my productivity.
Perceived ease of use (PEU)^a^	PEU1	My interaction with bracelet would be clear and understandable.
	PEU2	It would be easy for me to become skillful at using bracelet.
	PEU3	I would find bracelet easy to use.
Intrinsic sport motivation (ISM)^b^	ISM1	For the pleasure it gives me to know more about the sport that I practice.
	ISM2	For the pleasure of discovering new training techniques.
	ISM3	For the pleasure that I feel while learning training techniques that I have never tried before.
	ISM4	For the pleasure of discovering new performance strategies.
Extrinsic sport motivation (ESM)^b^	ESM1	Because, in my opinion, it is one of the best ways to meet people.
	ESM2	Because it is one of the best ways I have chosen to develop other aspects of myself.
	ESM3	Because it is a good way to learn lots of things which could be useful to me in other areas of my life.
	ESM4	Because it is one of the best ways to maintain good relationships with my friends.

*^a^Items were adapted from Davis (1989) and Davis et al. (1992).*

*^b^Items referred to Briére et al. (1995).*
